# Using the Welfare Quality® framework to develop a welfare assessment protocol for captive chimpanzees (*Pan troglodytes*)

**DOI:** 10.1017/awf.2025.10021

**Published:** 2025-07-29

**Authors:** Niamh Mc Gill, Miguel Bueno, Neil E. Anderson

**Affiliations:** 1 Dublin Zoo, Phoenix Park, Saint James, Dublin D08 WF88, The Republic of Ireland; 2 The Royal (Dick) School of Veterinary Studies and the Roslin Institute, University of Edinburgh, Roslin, UK

**Keywords:** Animal-based measures, animal welfare, chimpanzee, *Pan troglodytes*, welfare assessment, Welfare Quality®, zoo

## Abstract

Good welfare is of inherent value to all captive animals and promotes species conservation objectives. Concern for animal welfare is growing globally, and research shows that animal welfare is a top priority for zoo visitors. There is, therefore, an urgent need for zoos to develop and validate species-specific welfare assessment tools with a shift in focus away from avoiding negative affective states, and towards promoting positive ones. This shift in emphasis requires the development of comprehensive and robust welfare assessment protocols incorporating species-specific indicators. This study aimed to identify and propose welfare indicators for captive chimpanzees (*Pan troglodytes*) that could be used to adapt the EU Welfare Quality® protocol for this species. A literature review was carried out according to the Preferred Reporting Items for Systematic Reviews and Meta-analyses (PRISMA) guidelines and the authors followed the principles of a systematic review to identify a comprehensive set of welfare indicators for this species. Overall, 14 animal-based and 16 resource-based indicators are proposed to assess the 12 criteria and four principles of Welfare Quality®. This study represents the first effort to adapt the EU Welfare Quality® protocol to assess captive chimpanzee welfare and illustrates how this protocol can be adapted to develop a taxon-specific welfare assessment tool once species-specific natural history and biology are considered.

## Introduction

Global concern for the welfare of zoo animals has increased dramatically over the last decade, and animal welfare has now become a priority for modern zoos (Blokhuis 2008; Phillips *et al.* 2012). Despite a commitment to species conservation, it has been argued that housing wild animals in captive environments cannot be justified unless high welfare standards are achieved (Sherwen *et al.* 2018). To this end, formal evaluation of welfare status is now a core element of zoo accreditation schemes, such as that of the European Association of Zoos and Aquaria.

Animal welfare has been variably defined and is a multidimensional concept that encompasses an animal’s health, physiology, behaviour, and mental state (Mellor *et al.* 2009). Each of these dimensions subsumes several criteria; thus, animal welfare assessment poses a multicriteria evaluation challenge (Botreau *et al.* 2007). The Five Domains Model (Mellor & Reid 1994; Mellor & Beausoleil 2015) and Welfare Quality® protocol are two frameworks that have been used to assess the welfare of zoo animals and have become the foundation for developing multiple assessment tools in various captive species (McCulloch 2013). There have been several advances in animal welfare science over the past decade, with two of the most notable being the increasing focus on animal-based indicators and the promotion of a positive affective state (Jones *et al.* 2022). This shift in emphasis requires the development of comprehensive and robust welfare assessment protocols incorporating species-specific indicators.

Chimpanzees (*Pan troglodytes*) are now listed as ‘Endangered’ on the IUCN red list (Humle 2016), and both *in situ* and *ex situ* conservation efforts are increasingly crucial in ensuring stable wild populations and increasing public awareness of the global threats facing these primates. Assessing and improving their welfare in captivity is paramount for several reasons. Firstly, animals have an inherent right to good welfare and should be provided with environments that allow them to live according to their ecological niche and behavioural biology (Rose & Riley 2022). Humans, therefore, have a moral and ethical responsibility to offer animals the opportunity to experience ‘a good life’ as a fundamental need (Mellor 2016). Additionally, poor welfare can reduce an animal’s overall health and reproductive capacity, thus undermining conservation efforts. Captive animals with compromised welfare are also more likely to exhibit abnormal behaviours, thereby weakening their educational value for zoo visitors. (Skovlund *et al.* 2021).

The Welfare Quality® framework is one of the most widely accepted and comprehensive protocols for assessing animal welfare (Botreau *et al.* 2007). This framework was developed by a European research project that aimed to provide a standardised, science-based approach for evaluating the welfare of farm animals (Blokhuis 2013). Welfare Quality® uses four welfare principles, which reflect four primary areas of concern as agreed upon by the Welfare Quality® scientists. These principles are ‘Good feeding’, ‘Good housing’, ‘Good health’, and ‘Appropriate behaviour’, comparable to the four functional domains of the Five Domains model. Each of these principles comprises several more detailed aspects, and so two to four criteria were defined within each of the four principles, creating the twelve Welfare Quality® criteria that exist today. Great emphasis was placed on this set of criteria being exhaustive, minimal, readable, agreed upon by stakeholders, and independent of one another (Botreau *et al.* 2007). Welfare scientists also agreed early in the development process that these twelve welfare criteria should be applicable across all species and situations (Blokhuis 2013), although the relative importance of each would likely vary between species and circumstances. The Welfare Quality® protocol was chosen as the framework for this study due to its widely accepted structure, comprehensive design, and potential for application to other species in managed care settings (Clegg *et al.* 2015; Salas *et al.* 2018; Benn *et al.* 2019). As described by Mononen *et al.* (2012) in their adaptation of Welfare Quality® to farmed foxes (*Vulpes lagopus, V. vulpes*) and mink (*Neovison vison*), although some welfare indicators from the farm animal protocols may be suitable for many species, the most appropriate welfare indicators for each species must be identified. Botreau *et al.* (2012) emphasised the importance of the principles and criteria remaining consistent when applying this protocol to other species, and so the welfare assessment for captive chimpanzees incorporates all four principles and twelve criteria of Welfare Quality®. The current work builds upon the recent comprehensive review by Angley *et al.* (2024), which examined the relationship between the care provided to captive chimpanzees (resource-based indicators) and the resulting impact on their welfare (animal-based indicators).

Despite the need to assess captive chimpanzee welfare robustly and reliably, there have been few efforts to collectively define a set of validated indicators in this species. This study aimed to identify valid and feasible welfare indicators for captive chimpanzees and represents the first effort to adapt the Welfare Quality® protocol to this species.

## Materials and methods

### Introduction

A three-step process was employed in this study to adapt the Welfare Quality® protocol for captive chimpanzees:

- Step 1 – A brief review examined how this protocol was previously adapted for five other species in managed care;
- Step 2 – A literature review was conducted to identify a comprehensive list of welfare indicators for captive chimpanzees; and
- Step 3 – Valid, reliable, and feasible welfare indicators for chimpanzees were then assigned to each criterion of the Welfare Quality® protocol.

### Step 1 – Review of Welfare Quality® protocols adapted for other zoo species

Welfare Quality® has thus far been adapted for four other species in managed care: bottlenose dolphins (*Tursiops truncatus*) (Clegg *et al.* 2015); dorcas gazelles (*Gazella dorcas*) (Salas *et al.* 2018); pygmy blue-tongue skink (*Tiliqua adelaidensis*) (Benn *et al.* 2019); and farmed foxes and mink (Mononen *et al.* 2012). The first step in developing a protocol for captive chimpanzees was to review how this has already been adapted for other species. These Welfare Quality® protocols were reviewed, and the indicators chosen to assess each criterion were tabulated. The authors also chose to include the Welfare Quality® indicators outlined for dairy cattle (Welfare Quality® 2009) in this table, as the Welfare Quality® protocol developed for farm animal production served as the starting point for these adapted protocols. This review aimed to examine the developmental structure and considerations described by each author relevant to adapting the protocol to a novel species. Additionally, the authors sought to compare the indicators used to assess each criterion in these protocols to identify potential welfare indicators for consideration for the adapted Welfare Quality® protocol for captive chimpanzees.

### Step 2 – Identification of welfare indicators for chimpanzees

#### Search strategy

The second step in adapting this protocol for captive chimpanzees was to broadly review the literature to identify a comprehensive set of welfare indicators for this species. A literature review using a systematic methodology was undertaken in April 2022. This review was carried out according to the PRISMA (Preferred Reporting Items for Systematic Reviews and Meta-Analysis) guidelines and followed the principles of a systematic review to carry out an extensive search and apply inclusion and exclusion criteria. However, this review should not be considered ‘systematic’ as the authors worked alone under time constraints. Relevant studies were identified from searches of ‘all years’ on the following bibliographic databases:

- CAB Abstracts (OVID): 1973 – present;
- Medline (OVID): 1946 – present;
- Web of Science Core Collection (Web of Science): 1900 – present;
- BIOSIS Citation Index (Web of Science): 1926 – present;
- Zoological Record (Web of Science): 1864 – present; and
- Scopus (Scopus): 1788 – present.

A three-step search strategy was employed. Firstly, to develop a comprehensive search strategy, relevant keywords were identified by identifying and tabulating the keywords and index terms of relevant literature already in the authors’ possession. Scoping searches were then carried out to test these keywords and identify those which returned the most relevant results. Finally, searches were carried out using a combination of all identified and relevant keyword terms relating to captive chimpanzee welfare as outlined below (Table 1). The same keywords were used throughout all database searches, with minor variations using the relevant database thesaurus — MeSH or Index terms. Boolean operators (“OR,” “AND”) and truncation (*) were used to identify all relevant articles and records (see Appendix 1 in Supplementary material for bibliographic tables).

**Table 1.** Keywords used in searches of six reference databases to identify relevant articles to construct a comprehensive list of welfare indicators for captive chimpanzees

activit*	enclosure	human interaction*	quality of life	welfare
affiliativ*	enrichment	husbandry	relocat*	well-being
aggress*	groom*	injur*	sanctuar*	wound*
behav*	group size	mutilation	space	zoo*
captiv*	health*	pan troglodytes	stereotyp*	
chimpanzee*	housing	play*	stimulat*	
complexity	human animal relationship*	Posture	stress*	

#### Eligibility criteria

##### Inclusion criteria

Only publications that met all the following criteria were reviewed for the extraction of welfare indicators:

- the paper was specific to chimpanzees in captivity (to include those in sanctuaries, zoos, and laboratory settings), and chimpanzees were the principal subject of interest;
- the publication was available to the authors in full;
- the publication was in English;
- the publication was in a peer-reviewed journal; and
- the publication assessed the welfare, behaviour, physiology or physical condition of a captive chimpanzee at a point in time.

##### Exclusion criteria

Papers were excluded from the review if they did not meet all the above inclusion criteria or were review articles. Additionally, an exclusion criterion was applied to all studies examining infant and maternal behaviour, health, or nutrition, as although normal maternal and infant behaviour is highly relevant to welfare, these behaviours are likely to be specific to a particular life or developmental stage and are therefore not suitable as general welfare indicators. Finally, an exclusion was also applied to all studies relating to personality assessment due to the complexity of the analysis of such an indicator.

#### Study selection and indicator extraction

Search results were uploaded to Covidence systematic review software (Veritas Health Innovation, Melbourne, VIC, Australia), and duplicates were removed. The authors then used this software to screen the titles and abstracts of the studies for inclusion against the selection criteria outlined above and the full-text records sought for retrieval were then imported into EndNote 20 (Clarivate Analytics, PA, USA). These were then further assessed through full-text review, and literature not meeting all the eligibility criteria was excluded. The full texts of included articles were then reviewed and identified welfare indicators were captured and categorised using a table created in Microsoft Excel® (Microsoft® Corporation 2018).

#### Categorisation of indicators

Consistent with the Welfare Quality® approach, the primary aim in adapting this protocol for captive chimpanzees was to prioritise the use of animal-based (or welfare outcome-based) indicators over resource-based (or input-based) indicators, where feasible. Indicators identified from the literature search were therefore categorised as ‘animal-based’ or ‘resource-based’. Following on from the groupings adopted by Truelove *et al.* (2020) in their paper on laboratory-housed macaque (*Macaca mulatta*) welfare, indicators related to the environment, enrichment, and management practices were categorised as ‘resource-based’ indicators, while those related to the animal’s physical health, and the behavioural and physiological response to the environment were categorised as ‘animal-based’ indicators.

### Step 3 - Creation of a Welfare Quality® protocol for chimpanzees

All potential welfare indicators identified in Steps one and two were reviewed for validity, feasibility, and reliability by further consulting the literature identified in the review. In selecting the welfare indicators for captive chimpanzees, the authors assessed the relative strength of the available evidence for each indicator by evaluating factors such as the number of supporting studies, the research design, the statistical significance and effect sizes of the findings, and the consistency of results across publications. Indicators backed by a larger body of high-quality, consistent evidence were chosen over those with weaker or more conflicting support in the literature.

In line with Welfare Quality®, one primary aim in adapting this protocol for captive chimpanzees was to incorporate as many animal-based indicators as possible instead of resource-based ones. Where a valid, reliable, and feasible animal-based indicator could not be identified, a feasible input-based indicator that corresponded well with the animal-based indicators was chosen (Welfare Quality® 2009; pp 21–22). As mentioned, Botreau *et al.* (2012) emphasised the importance of the principles and criteria remaining consistent when applying this protocol to other species, and so the welfare assessment for captive chimpanzees incorporates all four principles and twelve criteria of Welfare Quality®. In their development of the C-Well protocol to assess captive bottlenose dolphin welfare, Clegg *et al.* (2015) adapted the Welfare Quality® criterion of ‘Ease of movement’ to instead focus on an ‘Appropriate environment’, and the authors of the current study have taken a similar approach. By renaming this criterion as ‘Appropriate environment’, the authors aim to capture a broader range of environmental and social factors that are particularly relevant to the welfare of captive chimpanzees, given their advanced cognitive and social capabilities. This shift in focus allows the adapted protocol to more comprehensively assess the suitability of the captive setting for meeting the complex needs of this endangered primate species.

## Results

### Step 1 - Review of Welfare Quality® protocols adapted for other zoo species

See Table 2.

**Table 2.** The Welfare Quality® protocols for five species in managed care were reviewed, and the indicators chosen to assess each criterion were tabulated below to identify common indicators used across all species

#	Welfare Quality® criteria:	WelFur (farmed Foxes & Mink)	C-Well (Bottle Nosed Dolphins)	Dorcas Gazelle	Pygmy Blue-Tongue Skink	Dairy Cattle
1.	Absence of Prolonged Hunger	Body condition score	Body condition score Frequency of weight measurements Dietary records	Body condition score	Body condition score Persistent food-seeking behaviour Food Intake Appropriate diet provided	Body condition score
2.	Absence of Prolonged Thirst	Cont. water availability Functioning & cleanliness of water points	Capillary refill time Hydration protocol	Number of water points Availability of water Cleanliness of water points	Signs of dehydration Unhurried drinking Cleanliness and availability for water	Water provision Cleanliness of water points Number of animals using H_2_O points
3.	Comfort Around Resting	Availability of a platform (Fox) Cleanliness of fur (Fox) Availability of a nest box (Mink) Resting quality of nest box (Mink)	Time budget	Not applicable	Animal cleanliness Enclosure cleanliness	Time to lie down Colliding with equipment Lying outside area Cleanliness of animals
4.	Thermal Comfort	Protection from exceptional weather conditions Bedding material and insulation of nest box (Mink)	Frequency of water temp. testing Water temp. & diet Shade	Availability of shade Availability of shelter	Basking (time budget) Appropriate thermal range Enclosure maintenance	Not applicable
5.	Ease of Movement (Changed to Appropriate Environment)	Space available for moving- area and height	(Criteria renamed ‘Appropriate Environment’) Echolocation Complexity of enclosure Swim speed & aerials Salinity & pH Coliform & chlorine Frequency of water quality testing Application of enrichment	Enclosure size Square metres available per animal	Stress-induced behaviours Enclosure size Movement into burrow Atypical locations Provision of burrow	Presence of tethering Access to outdoor pasture
6.	Absence of Injuries	Skin lesions of injuries Difficulty moving (Fox)	Total wound threshold Wounds from enclosure	Integument alterations Lameness	Wounds Lameness	Integument alterations Lameness
7.	Absence of Disease	Mortality Bent feet (Fox) Lameness (Mink) Ocular inflammation Impaired oral health (Fox) Diarrhoea Urinary Tract Infection (UTI) (Fox) Obviously sick animal	Frequency of cough (Respiration) Inhalation duration (Respiration) Discolouration (Eye) Squinting (Eye) Skin abnormalities (Skin) Mouth abnormalities (Skin) Blood parameters	Nasal discharge Ocular discharge Hampered respiration Diarrhoea	Musculoskeletal system Respiratory system Stomatitis Parasites	Coughing Nasal discharge Ocular discharge Hampered respiration Diarrhoea Vulvar discharge Milk Somatic Cell Count Mortality Dystocia Downer cow
8.	Absence of Pain Induced by Management Procedures	Use and type of neck tongs (Fox) Killing method	Blood draw Gastric tubing Voluntary restraint Emergency containment training	Not applicable	Access to vet treatment when needed	Disbudding Dehorning Tail docking Castration
9.	Expression of Social Behaviour	Social Housing Age at weaning and weaning procedure (Mink)	Presence of social behaviours	Affiliative behaviour Intra-specific aggression	Affiliative behaviour Co-occupant aggression Group size	Agonistic behaviour Cohesive behaviour
10.	Expression of Other Behaviour	Opportunity to use enrichment Opportunity to observe surroundings (Fox) Stereotypic behaviour Fur chewing	(Criteria renamed ‘Absence of Abnormal Behaviour’) Stereotypic behaviour	Stereotypic behaviour Enrichment programme	Hunting Interaction with a Transparent Boundary (ITB) Enrichment programme	Access to pasture
11.	Good HAR	Feeding test (Fox) Temperament test (Mink)	Response to trainer Non-food tactile interactions	Medical training programme Capture and handling	Human-directed aggression Capture restraint technique/conditioning	Avoidance distance
12.	Positive Emotional State	Temperament test Transportation of live animals	Not applicable	Not applicable	Exploratory behaviour	Qualitative Behavioural Assessment (QBA)

### Step 2 - Identification and categorisation of welfare indicators for chimpanzees

#### Literature review

**Figure 1.**
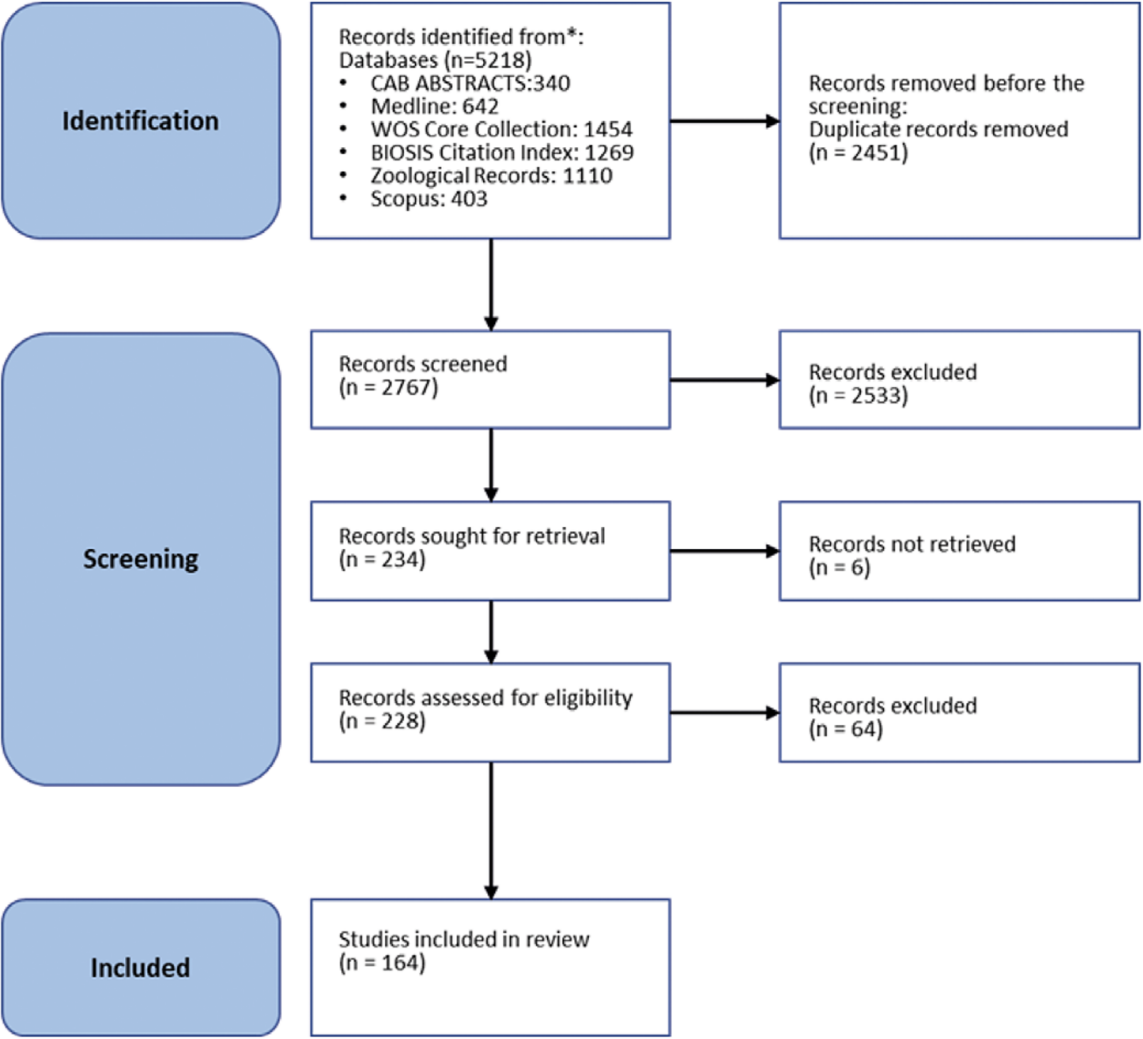
PRISMA 2020 flow diagram outlining the results of the sourcing of literature for the review of welfare assessment for captive chimpanzees (*Pan troglodytes*).

One hundred and one unique welfare indicators were extracted from 164 peer-reviewed papers Figure 1. The articles were published in 28 different journals, with most articles being found in *Zoo Biology, American Journal of Primatology, Applied Animal Behaviour Science*, *Animals*, *Primates*, and the *Journal of Applied Animal Welfare Science.* The articles were published by researchers in 13 countries, with 60% of the studies conducted by researchers in the USA. The included publications were published between 1982 and 2022.

#### Categorisation of indicators

A list of 101 potential indicators of captive chimpanzee welfare was generated, and each indicator was then categorised as an ‘animal-’ or ‘resource-based’ indicator. Indicators related to the environment, enrichment, and management practices were categorised as ‘resource’ (input) indicators, while those related to the animal’s physical health and the behavioural and physiological response to the environment were categorised as ‘animal’ (output) indicators. The 101 welfare indicators comprised 48 resource- and 53 animal-based indicators (Tables 3 and 4).

**Table 3.** Resource-based welfare indicators extracted from full texts of included studies were categorised as relating to the environment, enrichment or management practices

Environment	Enrichment	Management Practices
Access to outdoor space	Physical therapy programme	Attitude of keepers toward animals
Vertical space	Positive human interaction	Behavioural management programme
Auditory access to neighbouring groups	Occupational enrichment	Diet composition
Enclosure complexity	Cognitive enrichment	Number of times participated in a study
Enclosure size	Destructible enrichment	Positive reinforcement training
Enclosure type	Feeding enrichment	Levels of human activity
Environmental temperatures	Physical enrichment	Provision of dietary fibre
Escape opportunities	Sensory enrichment	Relocation
Group composition	Social enrichment	Transport
Group size	Provision of natural materials	Anaesthetic events
Naturalistic environment	Provision of nesting substrates	Spatial distribution of food
Provision of multilevel sleeping sites	Tactile communication	Temporal distribution of food
Provision of shade	Substrate type	Visitor numbers
Animal density		Preventative healthcare programme
Social housing		Meals per day: number
Spatial density		Meals per day: timing
Flooring type		
Ventilation

**Table 4.** Animal -based welfare indicators extracted from full texts of included studies were categorised as relating to health, behaviour or physiology

Health	Behaviour	Physiology
Body condition score	Abnormal behaviours	Biochemical values
Body Mass Index (BMI)	Affiliative behaviours	Neutrophil count
Bodyweight	Agonistic behaviours	Neutrophil to lymphocyte ratio
Weight gain	Inactivity	Platelet count
Obesity	Locomotion	Faecal glucocorticoid metabolites (FGM)
Discharge	Vocalisation	Hair cortisol
Cardiovascular health	Aggression towards caretaker	Urinary cortisol
Degenerative disease	Anxiety behaviours	Immunoglobulin-A
Growth rate	Behavioural diversity	Immunological values
Injuries (by conspecific)	Exploratory behaviour	Blood pressure
Injuries (not by conspecific)	Foraging behaviour	Acute phase proteins
Mobility/ Gait	Length of sleep period	
Parturition patterns	Nest building	
Periodontal health	Object manipulation	
Vitamin D deficiency	Positive interaction with humans	
Appetite	Presence of species-typical behaviour	
Coughing	Space-use	
Dyspnoea	Sleep quality	
Ocular discharge	Social stability	
Nasal discharge	Social vigilance	
Lethargy		
Mortality rate		
Urination, excessive or lack-of		

### Step 3 - Creation of a Welfare Quality® protocol for chimpanzees

The rationale for selecting the indicators used to evaluate each of the twelve criteria is explained in later sections. In total, 30 indicators were chosen, 14 of which were animal-based (47%), with eight of the twelve criteria including at least one animal-based indicator (Table 5).

**Table 5.** The 12 criteria and 30 indicators chosen to create the adapted Welfare Quality® protocol for captive chimpanzees

Principle		Criteria		Indicators
Good Feeding	C1.	Absence of Prolonged Hunger	1.1.	Body condition score (BCS) ^A^
			1.2.	Dietary records^R^
	C2.	Absence of Prolonged Thirst	2.1.	Number of water points ^R^
			2.2.	Continuous availability of water^R^
			2.3.	Cleanliness of water points^R^
Good Housing	C3.	Comfort Around Resting	3.1.	Provision of multilevel sleeping sites^R^
			3.2.	Provision of nesting materials^R^
			3.3.	Nesting behaviour^A^
	C4.	Thermal Comfort	4.1.	Availability of shelter and shade^R^
			4.2.	Environmental monitoring^R^
	C5.	Appropriate Environment	5.1.	Animal density^R^
			5.2.	Enclosure complexity^R^
			5.3.	Access to outdoor space^R^
Good Health	C6.	Absence of Injuries	6.1.	Wounding^A^
			6.2.	Social stability^R^
	C7.	Absence of Disease	7.1.	Cardiovascular health^A^
			7.2.	Signs of respiratory disease^A^
			7.3.	Signs of gastrointestinal disease^A^
			7.4.	Signs of musculoskeletal disease^A^
			7.5.	Signs of renal disease^A^
			7.6.	Preventative Healthcare Programme^R^
	C8.	Absence of Pain Induced by Management Procedures	8.1.	Behavioural Management Programme^R^
Appropriate behaviour	C9.	Expression of Social Behaviour	9.1.	Affiliative interactions^A^
			9.2.	Agonistic interactions^A^
			9.3.	Group size^R^
	C10.	Expression of Other Behaviour	10.1.	Abnormal behaviour^A^
			10.2.	Play & exploratory behaviour^A^
			10.3.	Enrichment programme^R^
	C11.	Good human-animal relationship (HAR)	11.1.	Response to a caregiver^A^
	C12.	Positive Emotional State	12.1.	Qualitative Behavioural Assessment (QBA)^A^

R Resource-based Indicator.

A Animal-based Indicator.

#### Good feeding

##### C1. Absence of prolonged hunger

Hunger is a subjective sensation of appetite and is difficult to assess in non-human animals, but well-designed indicators can suggest probable hunger or satiety (Dawkins 2006; Clegg *et al.* 2015). Following the Welfare Quality® model, the indicators proposed to assess this criterion and evaluate both the degree of detail included in dietary records as well as the body condition of the animal, as further discussed below.

The diet of captive chimpanzees has been associated with multiple conditions, including obesity, cardiac, dental, and gastrointestinal disease (Obanda *et al.* 2014), as well as contributing to abnormal behavioural repertoires, including regurgitation and reingestion (Cabana *et al.* 2018). The provision of a suitable diet, as assessed by dietary records, is thus of the utmost importance to captive chimpanzee management and encompasses both appropriate dietary composition and presentation. The nutrient composition of foods consumed by wild chimpanzees is high in fibre and low in water-soluble carbohydrates and lipids (Conklin-Brittain *et al.* 1998). Studies have demonstrated that as the proportion of dietary water-soluble carbohydrates decreases, the neutral detergent fibre (NDF) fraction increases, significantly influencing chimpanzee behaviour; inactivity and abnormal behaviour patterns decreased while locomotion, foraging, exploratory and affiliative social behaviours increased (Cabana *et al.* 2018). In addition to diet composition, food presentation is vital to captive chimpanzee management. Wild chimpanzees spend a large proportion of their time (22.5–30.7%) engaged in feeding and foraging behaviour (Hockings *et al.* 2009) and providing abundant foraging and feeding opportunities likely represents a source of social and cognitive stimulation, reducing inactivity and the performance of some abnormal behaviours (Celli *et al.* 2003). Research by Bloomsmith and Lambeth (1995) has also shown that unpredictable feeding schedules reduced inactivity levels before feeding while feeding whole items may increase feeding time and replicate wild manipulatory behaviours in chimpanzees. A good welfare score would thus be assigned if a chimpanzee’s dietary records reflected the current state of knowledge in this area. While this indicator does not directly assess the animal’s physical condition or feeding behaviour, it evaluates the comprehensiveness and quality of the dietary records maintained by the facility. Importantly, this indicator also serves to assess the appropriateness of the diet offered to the chimpanzees and looks for evidence that the diet provided is nutritionally appropriate and aligned with the natural foraging and feeding behaviours of chimpanzees.

Body Condition Scoring (BCS) is a well-established and widely used welfare tool that can be used to assess animal health. Obesity is a common problem when managing captive chimpanzees (Reamer *et al.* 2020), and a study by Obanda *et al.* (2014) highlighted similarities between humans and chimpanzees concerning obesity and the subsequent risk of developing inflammatory disease. Frequent assessment of body condition should therefore be an integral part of health management in captive chimpanzees, and caretakers should be able to quickly identify and assess overweight or obese individuals. Reamer *et al.* (2020) developed a ten-point scale for non-invasive observational ratings and this has been validated against actual weights and skin fold measurements from sedated chimpanzees.

##### C2. Absence of prolonged thirst

The continuous availability of water, the number of water-points, and the cleanliness of water-points were considered suitable indicators to assess this criterion, but they reflect the challenge in finding a feasible animal-based indicator (Botreau *et al.* 2009). Indicators to assess dehydration, such as plasma osmolarity, involve taking a blood sample and thus do not satisfy the requirements for a welfare assessment to be non-invasive, feasible to apply, and undemanding on resources (Jones *et al.* 2022). Chimpanzees should have easy access to multiple functioning water-points, and the number provided should consider the group size, social dynamics, and habitat design. Water-points should be cleaned regularly and free of crusts of dirt (for example, faeces) and decayed food residue with a good welfare score assigned where water is readily available in clean, functioning water-points to all troop members.

#### Good housing

##### C3. Comfort around resting

Chimpanzees build nests for sleeping by combining tree branches and vegetation (Goodall 1962; Koops *et al.* 2012), and these nests serve various functions, including shelter from climatic conditions, defence against predators, and comfort for sleep or rest (Pruetz *et al.* 2008; Stewart & Pruetz 2013). Captive chimpanzees may be provided with materials for nest-building, including hay, woodchips, blankets, and paper (Brent 1992), or with intact browse materials for constructing a more typical nest (Videan 2006). It is worth mentioning at this point that there is a need to distinguish between a behaviour that is ‘natural’ and one that is ‘motivated’ in an individual as there is limited evidence that ‘natural behaviours’ reliably either lead to or signify better well-being, unless an individual values that behaviour (Yeates 2018). For example, it is not expected that animals enjoy flight, sickness or antagonistic behaviours commonly encountered in ‘natural’ environments (Hermansen *et al.* 2004; Miao *et al.* 2004), while animals may find enjoyment in unnatural affiliative interactions with human caregivers (Yeates & Main 2008). Thus, it is impossible to determine *a priori* what ‘natural behaviours’ improve well-being (Yeates 2010).

Nesting behaviour forms a fundamental and valued part of a chimpanzee’s behavioural repertoire (Fruth & Hohmann 1996), and thus the provision of nesting materials has the potential to impact the welfare of captive chimpanzees. Multiple studies have demonstrated this, including Brent (1992), who found that supplying woodchip bedding to juvenile chimpanzees significantly reduced their abnormal behaviours. More recently, Anderson *et al.* (2021) demonstrated that an individual’s welfare was highest when presented with destructible, synthetic nesting materials. It is also worth noting that nest-building ability has been shown to depend on early rearing conditions and that bed-building techniques differ between wild- and captive-born chimpanzees (Bernstein 1962; Videan 2006).

Nests can be built on the ground or at a height, and so the provision of multilevel resting sites is the second indicator chosen to assess comfort around resting in this species. Night nests are typically constructed in a tree (Reynolds & Reynolds 1965), while day nests are often at ground level (Goodall 1962; Brownlow *et al.* 2001). Samson and Hunt (2014) investigated the physical comfort levels of tree and ground nests and reported that ground nests could be advantageous owing to reduced energy expenditure and a homeostatic microclimate. Chimpanzees construct a new nest each night, and a study by Lock and Anderson (2013) concluded that night-time spatial arrangements and individual preferences for specific sleeping areas were broadly comparable to nesting patterns reported in free-living chimpanzees. This study suggested that in the interests of captive ape welfare, habitats should include multilevel nesting areas and a choice of multiple sleeping sites.

##### C4. Thermal comfort

Chimpanzees inhabit various environments, from the semi-arid savanna to the warm and humid tropical forests (Pruetz & Bertolani 2007), implying behavioural flexibility to adjust to various habitats. Two indicators were developed to assess thermal comfort, adapted from the Welfare Quality® model: availability of shelter and shade and appropriate environmental monitoring records.

Chimpanzee activity budget, terrestriality, and sun exposure have been found to be related to environmental temperatures (Kosheleff & Anderson 2009). Studies investigating how chimpanzees vary their arboreal versus terrestrial budgets due to environmental temperatures have yielded conflicting results, with Kosheleff and Anderson (2009) concluding that chimpanzee terrestriality correlated negatively with temperature, while Takemoto (2004) found increased terrestrial activities with higher ambient temperatures. Nevertheless, it is apparent that chimpanzees exhibit behavioural flexibility and adapt to diverse conditions by employing various behavioural strategies for thermoregulation, allowing them to respond to thermal challenges associated with housing them away from their natural environment. Examples of such strategies come from studies such as that by Pruetz and Bertolani (2007), who demonstrated that savannah chimpanzees use caves to avoid heat in an exposed environment, while Duncan and Pillay (2013) concluded that chimpanzees in captivity adopted a sun-avoidance strategy, with ultraviolet radiation and humidity levels predicting shade utilisation patterns. Other studies assessing space use and environmental complexity have also demonstrated the tendency of captive apes to spend time close to buildings (Ogden *et al.* 1990), which may relate to the thermoregulatory benefits of microclimates around these structures (Stoinski *et al.* 2001). Thus, the best welfare score for thermal comfort would be assigned to enclosures with multiple shaded and sheltered areas available (for example, climbing structures and platforms), as these areas create a choice of differing microclimates for thermoregulation (Swaisgood & Shepherdson 2005).

In colder countries, captive chimpanzees may have to contend with low environmental temperatures and the provision of heated indoor enclosures and ‘hot spots’ are usually necessary. The highest welfare score for the second Welfare Quality® indicator relating to thermal comfort would evaluate environmental monitoring records and protocols to assess and prevent enclosure temperatures outside of chimpanzees’ Preferred Optimal Temperature Zone, with a remediation process outlined should temperatures fall outside of this range.

##### C5. Appropriate environment

This was the sole criterion altered from the Welfare Quality® protocol and was adapted to account for chimpanzees’ complex and advanced social structure and intellect. Like Clegg *et al.* (2015) in the adaption of Welfare Quality® for captive bottlenose dolphins, this criterion was changed from ‘Ease of movement’ to ‘Appropriate environment’.

There has been an increasing focus on enhancing functionally appropriate captive environments (FACEs) for chimpanzees, such that natural ranging behaviours and other valued species-typical behaviours are promoted (Webb *et al.* 2018a). Environments inhabited by wild animals often serve as the ‘gold standard’ when judging captive environments, and an abiding critique of captivity is the degree to which it differs to a wild environment, particularly concerning the space available for animals to range and travel. Animal density or space per animal is thus an essential focus of these FACEs, and studies on space per chimpanzee have demonstrated that higher animal densities are related to increased social tension and reduced allogrooming (Aureli & Waal 2000). Consequently, chimpanzees have been shown to use conflict-avoidance and tension-reduction strategies to cope with reduced space (Aureli & De Waal 1997). Chimpanzees have also been shown to engage in more abnormal and self-directed behaviours when confined at an increased density (Duncan *et al.* 2013). Thus, the best welfare score for spatial density would be assigned where a behaviourally relevant minimum size threshold was considered. This is supported by literature that suggests that welfare is enhanced when space is increased beyond a suboptimal minimum, with increasing space beyond this leading to smaller and smaller changes in behaviour and increases in welfare (Ross *et al.* 2011b; Reamer *et al.* 2015).

The topography of zoo enclosures has been associated with animal well-being, with novelty and enclosure complexity regarded as crucial to improving welfare (Jensvold *et al.* 2001; Hosey 2005). Such complexity is offered through spatial topography, such as vertical space (Caws *et al.* 2008), available spaces (Bettinger *et al.* 1994; Ross *et al.* 2011a; Herrelko *et al.* 2015), escape routes (Ross & Lukas 2006), and opportunities to exhibit control (Videan *et al.* 2005b; Bloomsmith *et al.* 2017; Webb *et al.* 2018b). Access to outdoor space is a further indicator purported to influence captive chimpanzee behaviour, as it results in significant volumetric expansion, increased naturalism, and increased enclosure complexity (Ross *et al.* 2011b). Thus, the appropriateness of the environment could be assessed by these three indicators, whereby more topographically complex and dynamic enclosures with access to outdoor space achieve better welfare scores.

#### Good health

##### C6. Absence of injuries

Socially housed chimpanzees are at risk of increased frequency of injury owing to intraspecific aggression, as exists in the wild. Therefore, captive management must balance the social needs of the animals with the desire to curtail wounding owing to natural intraspecific aggression (Williams *et al.* 2010). As agonistic behaviour is a natural part of the chimpanzee’s behavioural repertoire, its elimination from the captive setting is an unrealistic and perhaps undesirable objective. It is also worth noting that social conflict provides cognitive stimulation, variability, and problem-solving opportunities (de Waal *et al.* 1992). However, captive chimpanzees engage in more aggression than their wild counterparts, and a number of studies have identified factors that influence agonistic encounters and wounding in captivity, including: (1) introductions of new individuals (Alford *et al.* 1995); (2) the age and sex of the individual (Baker 2000); (3) group composition (Ross *et al.* 2009); (4) social stability (Bloomsmith *et al.* 1992); (5) the presence of in-oestrus females (Bloomsmith *et al.* 1992); and (6) the level of human activity (Lambeth *et al.* 1997). Management policies can be formulated to reduce the risk of serious wounding by identifying the main factors influencing wounding aggression in captive chimpanzees. The general recommendation that emerges from the literature cited above is that captive management should focus on maintaining long-term, stable social groups and minimise changes in group composition as Williams *et al.* (2010) demonstrated that wounding aggression decreased in groups with long-term social stability. Two indicators were chosen to assess the ‘Absence of injuries’ criterion, reflecting the main risk of injury to captive individuals, which is intraspecific wounding. The highest welfare score for the resource-based indicators would be assigned to a stable social grouping, while the highest score for the animal-based indicator would be assigned to a chimpanzee with no evidence of wounding.

##### C7. Absence of disease

Chimpanzees, like humans, can be affected by a wide range of diseases, and the Welfare Quality® indicators chosen to assess this criterion focus on the most prevalent chronic and acute diseases of captive chimpanzees.

The most frequent cause of death in captive chimpanzees has been repeatedly confirmed as cardiovascular disease (Kumar *et al.* 2017; Raindi *et al.* 2022; Ross *et al.* 2022), with a higher percentage of chimpanzees affected by this condition when compared to humans (Seiler *et al.* 2009). Interstitial fibrosing cardiomyopathy is the most common pathology identified, and sudden collapse and death are thought to result from defective conduction or sudden cardiac arrhythmia (Baldessari *et al.* 2013). Whereas humans typically suffer ischaemic myocardial injury due to occlusive vascular disease built up over a lifetime, chimpanzees develop myocardial fibrosis without the vascular component (Varki *et al.* 2009). Various initiatives, such as the Ape Heart Project based at Twycross Zoo and the International Primate Heart Project, have been established to further our understanding of heart disease in great apes, with causal factors remaining poorly understood despite extensive research (Strong *et al.* 2020).

Our understanding of what other diseases affect captive chimpanzees derives from various studies retrospectively examining mortality in captive chimpanzee groups over long periods. Ross *et al.* (2022) analysed the cause of death of 224 chimpanzees at 42 accredited zoos over 25 years and concluded that younger individuals are particularly at risk of trauma and infectious disease, while degenerative conditions continue to be the leading cause of death for adult chimpanzees. Ross *et al.* (2022) examined fifteen years of records relating to the deaths of 268 chimpanzees at a primate facility in New Mexico and determined that gastrointestinal disease was the primary cause of death in 41% of the cases there. Hubbard *et al.* (1991) and Kumar *et al.* (2017) reviewed post mortem examination records for a colony of chimpanzees at The Southwest Biomedical Foundation in Texas and identified cardiovascular, gastrointestinal, and respiratory disease as the leading causes of death in this colony. An additional study conducted by Nunamaker *et al.* (2012) sought to characterise the prevalence of chronic disease in a colony of sixteen geriatric female chimpanzees and documented a 23% incidence of renal disease. Thus, five animal-based indicators were chosen to assess an ‘Absence of disease’, reflecting the most commonly recognised health concerns of captive chimpanzees: cardiovascular disease, respiratory disease, gastrointestinal disease, musculoskeletal and renal disease. A single resource-based indicator reflects the formulation of a comprehensive preventative healthcare programme, with routine parasitology, regular review of medical histories, daily observations, and opportunistic physical and diagnostic examinations as core components of this.

##### C8. Absence of pain induced by management procedures

Welfare Quality® includes a criterion for assessing pain associated with management procedures, such as blood draws, injections, and capture for immobilisation. In addition, captive chimpanzees are commonly moved within and among enclosures for feeding, training, veterinary care, or in emergencies where rapid access or separation is needed; thus, the ability to achieve this with voluntary animal participation is critical to animal health, safety, and welfare. The indicator proposed to assess this criterion is whether a behavioural management programme (BMP), such as positive reinforcement training (PRT), is in operation.

Eliciting co-operation from captive animals during necessary routine procedures through PRT is desirable, rather than forcing compliance using restraints or aversive measures (Reinhardt 2003). Multiple studies have now been published which have variably focused on training captive chimpanzees to co-operate during routine medical procedures (Videan *et al.* 2005a), moving cages (Bloomsmith *et al.* 1998), blood and urine collection (Schapiro *et al.* 2003; Reamer *et al.* 2014; Bloomsmith *et al.* 2015), as well as behavioural management that can reduce social tension during feeding (Bloomsmith *et al.* 1994). In addition to the benefits offered to the animal in terms of stress reduction, the involuntary collection of blood from distressed animals can result in blood samples that are not physiologically representative of the animal’s normal hormone levels during stress-free periods (Hassimoto *et al.* 2004). A study by Lambeth *et al.* (2006) also demonstrated that chimpanzees trained to present for an anaesthetic injection had lower white blood cell counts and blood glucose levels, indicating a reduced physiological stress response. Thus, PRT programmes allow animals to participate actively in interactions that improve veterinary care (Fernandez 2022).

PRT has additionally been shown to benefit animals by influencing social interactions with conspecifics, serving as a form of enrichment, and reducing undesirable behaviours. Bloomsmith *et al.* (1994) effectively reduced aggression during feeding times using PRT in group-housed chimpanzees. Pomerantz and Terkel (2009) used PRT to increase chimpanzee affiliative behaviours outside of training sessions, while they also demonstrated reduced abnormal and stress-related behaviours during these PRT sessions. Thus, the highest welfare scores for this criterion would be assigned to facilities with an established and active behavioural management programme in operation.

#### Appropriate behaviour

##### C9. Expression of social behaviours

Chimpanzees live in large, highly complex, multi-male, multi-female social communities with fission-fusion dynamics (Goodall 1986). Thus, they require social interactions with conspecifics to promote the expression of natural social behaviour. Group dynamics are somewhat artificial in captivity due to behavioural management programmes, operational decisions regarding housing, and the limited scope for escape or avoidance. Therefore, the indicators chosen to assess this criterion included group size and systematically measuring for agonistic and affiliative behaviours, like those exhibited by wild chimpanzees.

Humane standards for the long-term maintenance of captive chimpanzees mandate that they are socially housed, with a multitude of studies demonstrating that aggression and abnormal behaviours are higher in socially impoverished conditions (Spijkerman *et al.* 1994; Williams *et al.* 2010; Khan 2013). In terms of group size, the National Institutes of Health (NIH) and the Association of Zoos and Aquariums (AZA) recommend that captive chimpanzees are housed in multi-male, multi-female, age-diverse groups of no less than seven individuals (NIH 2013). Neal Webb *et al.* (2019) investigated this recommendation by examining the behavioural effects of group size, group age, and percentage of males on the group and found evidence to support the existing group size and composition recommendations. They also demonstrated in this study that chimpanzees living in more age-diverse social groups, with at least 50% males, showed the highest levels of affiliation.

It has been suggested that behavioural assessment, specifically social interactions, may inform welfare assessment more than physiological indicators (Hill & Broom 2009). The comparison of captive chimpanzee behaviour to that of wild conspecifics is often used as a proxy for animal welfare assessment, although Howell and Cheyne (2019) emphasise that all behaviours are stimulus-driven and that good welfare does not necessarily mean that behavioural budgets must reflect those of wild populations. A better approach may be determining if the animal is displaying affiliative behaviours such as allogrooming and social play, indicative of a positive emotional state (Boissy *et al.* 2007). Boissy *et al.* (2007) suggest that such behaviours may help infer good welfare as they often occur only when other physical needs are met. Furthermore, affiliative interactions have been shown to help alleviate stress in primates (Aureli & Yates 2010), while Yamanashi *et al.* (2018) demonstrated how adult-adult social play was used to reduce social tension in multi-male groups.

Conversely, there is little evidence to suggest the ‘normal’ frequency of agonistic behaviour among chimpanzees, but multiple studies demonstrate declining welfare states with the expression of agonistic behaviour in high frequencies (Alford *et al.* 1995; Videan & Fritz 2007; Ross *et al.* 2009; Fultz *et al.* 2015; Hosey *et al.* 2016). Intra-specific aggression also often leads to injuries and social stress and has therefore been included to assess this criterion in all the adapted zoo animal Welfare Quality® protocols outlined in Table 2. While agonistic behaviour is an expected and natural part of the chimpanzee’s behavioural repertoire, a higher welfare score would be assigned where agonistic behaviour is observed at lower frequencies.

##### C10. Expression of other behaviours

For captive chimpanzees, ‘abnormal behaviours’ are considered species-atypical behaviours that are observed either exclusively in captivity or at higher rates in captivity than in the wild (Hopper *et al.* 2016a). Both types are used as benchmarks for evaluating captive chimpanzee welfare, and several stereotypic behaviours have been empirically linked to elevated stress and suboptimal social and physical environments in primates, thus validating them as reliable indicators of poor welfare (Garner 2005). A study by Birkett and Newton-Fisher (2011) examined the behaviour of forty socially housed captive chimpanzees from six collections in the USA and the UK and concluded that abnormal behaviour is endemic in captive chimpanzee populations, despite enrichment efforts. Spijkerman *et al.* (1994) followed the behavioural development of ninety chimpanzees and demonstrated that body rocking resulted from the inability to cope with frustrating social circumstances, as well as early maternal separation. Baker and Aureli (1997) demonstrated that yawning, a self-directed behaviour related to anxiety, increased during periods of social tension in captive chimpanzees, while self-scratching has been observed to increase in chimpanzees in stressful situations, such as immediately after a conflict (Leavens *et al.* 2004; Koski & Sterck 2007), and to decrease after reconciliation (Fraser *et al.* 2008). However, it is worth noting that a clear link between abnormal behaviour and compromised welfare cannot be found in all studies. Recent research on coprophagy (deliberately eating faeces) by both Jacobson *et al.* (2016b) and Hopper *et al.* (2016a), suggested an ambiguous relationship between this ‘abnormal behaviour’ and welfare, as it is associated more frequently with desirable conditions, such as mother-rearing. The first Welfare Quality® indicator chosen to assess the ‘expression of other behaviour’ criterion assesses for the presence or absence of ‘abnormal’ behaviours, with the highest welfare scores assigned to animals not displaying species-atypical behaviour at the time of assessment.

As discussed in the *Introduction*, welfare science has shifted away from the avoidance of negative affective states, towards the promotion of positive ones (Yeates & Main 2008; Mellor 2012), with Boissy *et al.* (2007) even suggesting that the presence of positive affective states may be more relevant to welfare assessment. Therefore, the second indicator chosen to assess this criterion looks for the presence of ‘play and exploratory behaviours’, thus acknowledging the need for new welfare assessment tools to accommodate this shift and include positive welfare indicators where possible. Play behaviour is a valid indicator of positive welfare as it only occurs when all other needs are met (Held & Špinka 2011), while curiosity-driven exploration is a goal-directed behaviour, proposed to be indicative of a positive affective state (Mellor 2015a,b).

The operation of an environmental enrichment strategy was chosen as the third indicator to assess this criterion. Chimpanzees are highly social and cognitively complex animals, often living in environments that differ substantially from their wild counterparts, and thus appropriate enrichment is required to ensure their psychological well-being (de Groot & Cheyne 2016). The provision of enrichment seeks to encourage behaviours indicative of positive welfare, such as play, affiliation, and exploration (Boissy *et al.* 2007) while either reducing or decreasing the occurrence of negative behaviours (Tarou & Bashaw 2007; Wallace *et al.* 2017). Such enrichment can be sensory (Grunauer & Walguarnery 2018; Obsen *et al.* 2021), tactile (Cerrone 2019), cognitive (Yamanashi *et al.* 2016), physical (Hopper *et al.* 2016b), or food-related (Padrell *et al.* 2021).

##### C11. Good human-animal relationship

The human-animal relationship (HAR) was outlined by Hosey (2008) as the history of interactions between the human and the animal, which allows each to make predictions about the behaviour of the other. Negative interactions are generally aversive, unpredictable, and rough (Waiblinger *et al.* 2006), and Hosey and Melfi (2015) highlighted the negative impact of such interactions on zoo animal welfare. They demonstrated the negative impact of keepers with deficient handling skills and the stressing consequences of poor human-animal relationships when multiple keepers care for the same animal. Conversely, recent work has shown that positively reinforcing and affiliative relationships can enhance welfare, established through positive reinforcement training, considerate handling, familiarity, and increased interactions (Hosey 2008; Brando 2012). Positive interactions involve reassuring verbal and considered physical effort (Hemsworth 2003), and a significant body of evidence shows that positive human-animal relationships broadly improve captive chimpanzee welfare (Jensvold 2008; Chelluri *et al.* 2013). Baker (2004) demonstrated how increased human interaction led to increased affiliative encounters and reduced abnormal behaviours in a group of zoo-housed chimpanzees, while multiple authors have proposed that these positive interactions can further serve as social enrichment with positive welfare benefits (Jensvold *et al.* 2010; Claxton 2011; Chelluri *et al.* 2013).

Chimpanzees actively participate in daily husbandry schedules, such as shifting enclosures to facilitate cleaning, and these frequent animal-keeper interactions make HAR assessment particularly pertinent as a welfare metric, with two indicators chosen to assess this criterion for Welfare Quality®. The indicator proposed to assess this criterion is the response of a chimpanzee to a caregiver when not under stimulus control, with the highest welfare scores assigned to a positive response and the lowest welfare score assigned in cases of apparent fear avoidance, stress, or overt human-directed aggression.

##### C12. Positive emotional state

Increasing focus on the area of positive emotions, in combination with the assertion that captive wild animals should experience good welfare and ‘a good life’ (Mellor 2016), is leading to a greater aspiration among welfare scientists to promote positive experiences and enhance long-term positive emotional states. However, as highlighted by Boissy *et al.* (2007), such positive emotional states are usually more subtle, often less expressive, and more difficult to reliably distinguish when compared to negative ones. Qualitative Behavioural Assessment (QBA) is a scientific method that was initially developed by Wemelsfelder *et al.* (2000, 2001) and has been validated to contribute to the identification of the main dimensions of animal emotional states (Mendl *et al.* 2010; Rutherford *et al.* 2012; Temple *et al.* 2013; Carreras *et al.* 2016). This tool captures the expressive qualities of animal behaviour by observing an animal’s body language and then scores several behavioural descriptors such as ‘tense’, ‘calm’, ‘playful’, or ‘fearful’. The emotional connotations of these behavioural terms directly relate to animal welfare in that they describe the animals’ experience of their own state (Wemelsfelder *et al.* 2001; Wemelsfelder & Farish 2004). Ultimately, this method enables a highly experienced caretaker to capture subtle shifts in behaviour, posture, attitude, expression, or movement and to translate them into quantitative measures that can be analysed statistically.

## Discussion

### Assigning indicators to the Welfare Quality® criteria

The use of animal-based indicators is preferred over resource-based indicators, as these are assumed to possess a higher validity, given that they are more closely related to the current welfare state of the animal (Blokhuis *et al.* 2010). The time required to assess animal-based indicators is, however, their primary constraint concerning feasibility (Mulleder *et al.* 2007; Knierim & Winckler 2009). This is exemplified by the Welfare Quality® protocol for dairy cattle, where 60% of the indicators are animal-based but take about 90% of the total on-farm assessment time (de Vries *et al.* 2013).

As already described, welfare assessment tools should be feasible to apply, non-invasive, and undemanding on resources (Jones *et al.* 2022). Thus, a welfare assessment tool must strike a balance by incorporating as many animal-based indicators as possible while also including more feasible resource-based indicators that have been validated as reflective of an animal’s welfare state at a point in time (Truelove *et al.* 2020). As stated by Wolfensohn *et al.* (2018), from a practical viewpoint, a combination of both resource- and animal-based indicators is likely to promote a more comprehensive assessment of animal welfare while being reasonably practical to implement. Of the 30 indicators chosen for inclusion in the adapted protocol for captive chimpanzees, 14 are animal-based (47%), with eight of the twelve criteria including at least one animal-based indicator. The authors chose exclusively resource-based indicators for the remaining four criteria for reasons relating to the feasibility and validity of the considered animal-based indicators as outlined below.

The second Welfare Quality® criterion focuses on the ‘Absence of prolonged thirst’ and animal-based indicators which were considered to assess this criterion include the ‘pinch test’, the appearance of sunken eyes, or the collection of a blood sample for plasma osmolarity and haematocrit counts. The ‘pinch test’ on a captive chimpanzee would require anaesthesia for handling, so this was excluded for reasons relating to feasibility. The appearance of sunken eyes can only be used to detect dehydration in extreme cases and thus is not appropriate as an indicator to detect the suboptimal provision of water. Finally, plasma osmolarity and haematocrit counts increase when animals are deprived of water (Knowles *et al.* 1995; Pritchard *et al.* 2006), but their assessment requires the collection of a blood sample which is not consistent with a non-invasive welfare assessment. Thus, favouring resource-based indicators to assess this criterion was justified, given the lack of a well-validated, feasible animal-based alternative.

The indicators proposed to assess the three criteria of ‘Good housing’ were also exclusively resource-based in the adapted chimpanzee Welfare Quality® protocol. For the ‘Thermal comfort’ criterion, animal-based indicators, such as visible shivering and panting, could be regarded as valid signs of compromised welfare, but these indicators depend on the ambient temperatures at the time of assessment and, as such, do not reflect an ongoing welfare state. Consequently, the indicators chosen to assess this criterion focus on offering multiple opportunities for animals to maximise their thermal comfort in extreme weather conditions (i.e. the provision of shade and shelter), as well as an environmental monitoring policy that ensures temperatures in indoor habitats remain in the animals Preferred Optimal Temperature Zone. Similarly, for the ‘Comfort around resting’ criterion, an animal-based indicator related to sleep duration and quality could have been chosen, but again the assessment of this indicator is inconsistent with the stated aims of a welfare assessment tool being feasible and undemanding on resources (Jones *et al.* 2022).

Additionally, exclusively resource-based indicators were chosen to assess the ‘Appropriate environment’ criterion, although it is worth mentioning that some overlap exists between this criterion and the ‘Expression of social behaviour’ and ‘Expression of other behaviour’ criteria. The ‘Appropriate environment’ relates to the welfare ‘input’, whereby the expression of behaviours is the welfare ‘outcome’ in many situations pertinent to chimpanzee welfare. We know from the literature that abnormal behaviours have been linked empirically to suboptimal physical environments in primates (Nash *et al.* 1999; Garner 2005; Birkett & Newton-Fisher 2011), while the expression of social behaviours is directly correlated with social density (Webb *et al.* 2019) and space allowance (Neal Webb *et al.* 2018). Thus, resource-based ‘input’ indicators to assess the environment were assigned to ‘Appropriate environment’ while the ‘welfare outcome’ indicators of behaviour were assessed under the criteria examining the expression of social and other behaviours. Although it could be argued that this contradicts the aim of Welfare Quality® to have minimal and mutually independent criteria, all three criteria were included as there are many situations whereby the expression of social and other behaviours in this species may relate to factors unconnected to the environment (for example, the presence of new personnel).

Finally, the authors acknowledge that the strategy of relying upon comprehensive record and management programme evaluations as indicators may introduce some limitations in terms of practicality and accuracy, compared to more direct animal-based measures. However, these indicators can still provide valuable insights into how well the captive chimpanzees are cared for, which has been shown in the literature to have a direct impact on their welfare. For example, numerous studies have demonstrated that the quality and appropriateness of the diet provided, as reflected in dietary records, can significantly influence the physical health, behaviour, and overall welfare of chimpanzees in captivity (Conklin-Brittain *et al.* 1998; Cabana *et al.* 2018). Similarly, the presence and implementation of a comprehensive behavioural management programme, including positive reinforcement training, has been linked to reduced stress, increased affiliative interactions, and other indicators of positive welfare in this species (Bloomsmith *et al.* 1994; Pomerantz & Terkel 2009). By evaluating the content and implementation of these care-related records and programmes, the welfare assessment protocol can provide a more holistic understanding of the captive environment and management practices, and how they contribute to the animals’ overall well-being.

### The use of ‘abnormal behaviour’ as a welfare indicator

It is worth also discussing the use of ‘abnormal behaviour’ as an indicator of negative welfare, as this relationship is often complicated due to the complex mechanisms underlying many stereotypies (Jacobson *et al.* 2016b). Mason and Latham (2004) suggest that these abnormal behaviours can reflect behavioural flexibility, as animals attempt to satisfy a motivation to perform natural behaviours in captivity. They also suggest that, over time, this behaviour could be reduced to a habit that is no longer linked to the harmful context during which it developed, thus not necessarily reflecting the current welfare state of an animal. Additionally, certain stereotypic behaviours are thought to be spread via social learning (Jacobson *et al.* 2016a), with factors such as relatedness and rank implicated (Hopper *et al.* 2016a). Recent research on coprophagy by Hopper *et al.* (2016a) suggests its link to poor welfare is ambiguous, while the classification of self-plucking and ‘regurgitation and reingestion’ as ‘stereotypic’ behaviours is also contested (Baker & Easley 1996; Hosey & Skyner 2007). Thus, the extent to which chimpanzee behaviours deemed as ‘abnormal’ are actual manifestations of stress is debated, and a holistic understanding of the aetiologies of these behaviours is critical to determining whether they can be used as valid and reliable indicators of compromised welfare in captive chimpanzees. Furthermore, it highlights the importance of understanding the individual’s abnormal behavioural repertoire as if individuals can reliably vary in this repertoire, it seems plausible that such variation may also be apparent in the abnormal behaviours linked to behavioural signals of stress.

### Scoring system

**Figure 2.**
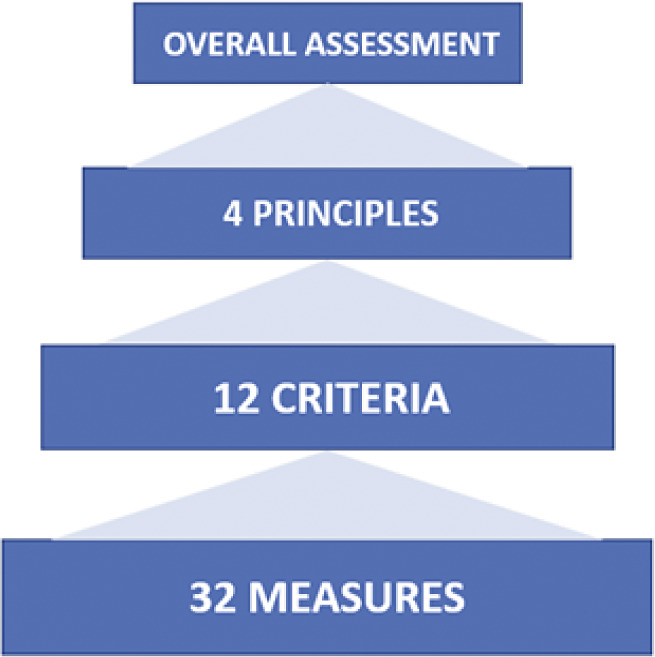
The bottom-up approach of the Welfare Quality® scoring system, with aggregation of welfare indicators into 12 criteria scores, grouped further into four principle scores, and finally into an overall welfare categorisation.

The Welfare Quality® scoring system requires the sequential aggregation of welfare indicators into 12 criteria scores, grouped further into four principle scores, and finally into an overall welfare categorisation as outlined in Figure 2. This scoring is a highly complex task for several reasons. Firstly, scoring the various indicators requires interpretation and balancing, and a degree of subjectivity is inevitable when weighting their relative importance (Spoolder *et al.* 2003). De Graaf *et al.* (2017) looked at the sensitivity of the integrated Welfare Quality® scores in dairy cattle and suggested that the integration method of this protocol be revised to ensure that the relative contribution of the welfare indicators to the integrated scores more accurately reflected their relevance for dairy cattle welfare. Secondly, a decision must be made regarding whether compensation between criteria is allowed, such that good scores on specific criteria can compensate for bad scores on others (Botreau *et al.* 2007). Thirdly, a scoring system must balance society’s expectation of welfare standards with the expert opinion of animal scientists and what is achievable in practice. Finally, it has been argued that value judgements are inherently involved when performing overall welfare assessments (Fraser 1995), although others claim that overall welfare assessment is not arbitrary and that a high level of accuracy can be achieved (Bracke *et al.* 1999). Thus, careful consideration must be given to the weighted scoring and aggregation system assigned to the proposed Welfare Quality® protocol for chimpanzees for meaningful overall welfare assessments to be produced.

### Limitations and recommendations for future research

This study has four main limitations. The first limitation relates to the approach used to identify relevant literature. Literature reviews are current only at the point in time at which they are conducted, and work published post the search date is excluded. Additionally, this review focused only on peer-reviewed literature, although ‘grey literature’ would likely have offered valuable information from relevant within-zoo studies. Limiting the searches to the English language and articles available to the authors in full at the time of the search also potentially excluded relevant literature. The second limitation relates to time constraints. Given the vast amount of literature published on captive chimpanzee welfare, a decision was made to extract indicators from all included studies without a prior critical appraisal of each paper. While the validity of the indicators chosen was informally appraised prior to their inclusion in the adapted protocol, a critical appraisal of all the included studies using a specifically designed welfare indicator appraisal tool (similar to the one used by Williams *et al.* 2018) would have further validated the chosen indicators. The authors acknowledge the limitations inherent in this retrospective, literature-based approach to indicator selection. Future efforts to implement this welfare assessment protocol should include a more systematic evaluation of the strength of evidence supporting each indicator to further strengthen the validity and reliability of the final tool. The third limitation of this study is that the lead author (NM) worked alone in reviewing the literature. Although the literature review followed a systematic methodology, it was not considered a ‘systematic review’ as reviews carried out by one person can inadvertently lead to increased bias in searching, screening, and data selection, as well as an increased chance of errors being made. The final limitation of this study is that the authors did not consult expert opinion before proposing indicators for this adapted Welfare Quality® protocol. Used in conjunction with one another, reviews of the existing literature alongside consultation with experts (keepers and specialists) would ensure that this adapted Welfare Quality® protocol offered a widely accepted and comprehensive welfare assessment tool.

In future studies, it would be useful to expand the research team and formally appraise the evidence from peer-reviewed literature on potential welfare indicators for captive chimpanzees. Subsequently, a workshop could be scheduled to refine the proposed indicators and design a scoring system that determines how indicators and criteria are scored, weighted, and aggregated into an overall welfare assessment. Finally, efforts could focus on piloting a functional welfare assessment tool, with subsequent re-evaluation and fine-tuning as needed.

### Animal welfare implications

This study represents the first effort to adapt the Welfare Quality® protocol to assess captive chimpanzee welfare, and the methodology used for this study can be applied to other species in managed care. Developing species-specific welfare assessment protocols such as this contributes to the advancement of robust zoo animal welfare assessment methods at a time when both *in situ* and *ex situ* conservation efforts are increasingly crucial.

Additionally, the current manuscript builds upon the recent comprehensive review by Angley *et al.* (2024), which examined the relationship between the care provided to captive chimpanzees (resource-based indicators) and the resulting impact on their welfare (animal-based indicators). The Angley *et al.* review served as the foundation for the development of the ChimpCARE Tool, an online resource that integrates the scoring, weighting, and aggregation of various welfare indicators to assess the well-being of captive chimpanzees.

While the ChimpCARE Tool was not specifically designed within the Welfare Quality® framework, the authors recognise substantial overlap between the indicators included in the current manuscript and those utilised in the ChimpCARE Tool. Both resources emphasise the importance of combining measures of the captive environment and management practices with direct assessments of the animals’ physical, behavioural, and mental states to comprehensively evaluate chimpanzee welfare.

The current work expands upon the literature synthesis provided in the Angley *et al.* review by specifically identifying and proposing a comprehensive set of welfare indicators for captive chimpanzees. Furthermore, by adapting the Welfare Quality® protocol for captive chimpanzees, the current manuscript offers an alternative, standardised framework for welfare assessment that can be compared to and potentially integrated with the ChimpCARE tool. The authors believe that the integration of these various perspectives and methodologies will provide caregivers and researchers with a suite of complementary resources to advance the assessment and improvement of welfare for this endangered primate species.

## Conclusion

In conclusion, this work represents the first effort to adapt the EU Welfare Quality® protocol to assess captive chimpanzee welfare by proposing a set of valid, feasible, and reliable welfare indicators for this species. The proposed protocol was developed after substantial consultation with the published literature, and 14 (47%) animal-based and 16 (53%) resource-based welfare indicators were included to assess the 12 criteria and 4 principles of Welfare Quality®. This adapted protocol also includes indicators of positive affective state, reflecting the aim of modern welfare assessment tools to accommodate the shift in emphasis from avoiding negative affective states to promoting positive ones. Improving animal welfare standards has now become an institutional priority in the zoo industry, and thus the first effort to develop this chimpanzee-specific welfare assessment protocol will begin to enable zoos to make evidence-based assessments meaningful for this species.

## Supporting information

Mc Gill et al. supplementary material Mc Gill et al. supplementary material

## Supplementary material

The supplementary material for this article can be found at http://doi.org/10.1017/awf.2025.10021.
