# Supplementary material for: Using the Welfare Quality® framework to develop a welfare assessment protocol for captive chimpanzees (Pan troglodytes)
Source: Anim Welf. 2025 Jul 29;34:e54. doi: 10.1017/awf.2025.10021 (PMC12304783; doi:10.1017/awf.2025.10021)
Supplement: Mc Gill et al. supplementary material [file S0962728625100213sup001.pdf]

Using the Welfare Quality® framework to develop a welfare assessment protocol for captive chimpanzees (*Pan troglodytes*)

Niamh McGillhttps://orcid.org/0009-0007-0296-219X[1], Neil E Anderson[2] and Miguel Bueno[1]

[1] Dublin Zoo, Phoenix Park, Saint James, Dublin D08 AC98, The Republic of Ireland

[2] The University of Edinburgh, Royal Dick School of Veterinary Studies, Roslin, Midlothian, UK

Author for correspondence: Neil E Anderson, email: neil.anderson@ed.ac.uk

Appendix

1.1. Search Summary

Database Name	Platform	Date Coverage	Date of Final Search	# of results
1. CAB ABSTRACTS	OVID	1973- present	13/02/2022	340
2. MEDLINE	OVID	1946- present	13/02/2022	642
3.WOS Core Collection	Web of Science	1900- present	13/02/2022	1454
4. BIOSIS Citation Index	Web of Science	1926- present	13/02/2022	1269
5. Zoological Record	Web of Science	1864- present	13/02/2022	1110
6. Scopus	Scopus	1788- present	13/02/2020	403

Total Records = 5218

Total Records after deduplication = 2767

1.2. Primary Search Strategy

1. CAB Abstracts (OVID)

Date of Final Search: 15/04/2022

Number of results: 340

Name: FULL SEARCH CAB FINAL V7_CAPTIVE_CHIMP

#	Search string	# of results
1	zoo animals.sh.	13708
2	captiv*.mp. [mp=abstract, title, original title, broad terms, heading words, identifiers, cabicodes]	22419
3	zoo*.mp. [mp=abstract, title, original title, broad terms, heading words, identifiers, cabicodes]	132655
4	sanctuar*.mp. [mp=abstract, title, original title, broad terms, heading words, identifiers, cabicodes]	3597
5	chimpanzees/	1852
6	chimpanzee*.mp. [mp=abstract, title, original title, broad terms, heading words, identifiers, cabicodes]	2415
7	pan troglodytes.mp.	844
8	1 or 2 or 3 or 4	151102
9	5 or 6 or 7	2433
10	8 and 9	785
11	animal welfare/	39858
12	welfare.mp. [mp=abstract, title, original title, broad terms, heading words, identifiers, cabicodes]	70576
13	stress.sh.	158725
14	stress*.mp. [mp=abstract, title, original title, broad terms, heading words, identifiers, cabicodes]	471452
15	animal health/	31360
16	health*.mp. [mp=abstract, title, original title, broad terms, heading words, identifiers, cabicodes]	966003
17	"quality of life"/	18982
18	("quality of life" or quality-of-life).mp. [mp=abstract, title, original title, broad terms, heading words, identifiers, cabicodes]	31079
19	enrichment/	11133

20	enrichment.mp. [mp=abstract, title, original title, broad terms, heading words, identifiers, cabicodes]	74748
21	animal husbandry.sh.	14851
22	husbandry.mp. [mp=abstract, title, original title, broad terms, heading words, identifiers, cabicodes]	39322
23	exp animal behaviour/	90802
24	behavio*.mp. [mp=abstract, title, original title, broad terms, heading words, identifiers, cabicodes]	552350
25	exp aggression/	5128
26	aggress*.mp. [mp=abstract, title, original title, broad terms, heading words, identifiers, cabicodes]	40239
27	injur*.mp. [mp=abstract, title, original title, broad terms, heading words, identifiers, cabicodes]	146313
28	wound*.mp. [mp=abstract, title, original title, broad terms, heading words, identifiers, cabicodes]	45859
29	(f?ecal adj3 glucocorticoid adj3 metabolite*).mp. [mp=abstract, title, original title, broad terms, heading words, identifiers, cabicodes]	257
30	grooming/	1096
31	groom*.mp. [mp=abstract, title, original title, broad terms, heading words, identifiers, cabicodes]	3138
32	stereotyp*.mp. [mp=abstract, title, original title, broad terms, heading words, identifiers, cabicodes]	4745
33	mutilation.mp. [mp=abstract, title, original title, broad terms, heading words, identifiers, cabicodes]	689
34	complexity.mp. [mp=abstract, title, original title, broad terms, heading words, identifiers, cabicodes]	47513
35	enclosure.mp. [mp=abstract, title, original title, broad terms, heading words, identifiers, cabicodes]	4165
36	relocat*.mp. [mp=abstract, title, original title, broad terms, heading words, identifiers, cabicodes]	5201
37	affiliative.mp. [mp=abstract, title, original title, broad terms, heading words, identifiers, cabicodes]	408
38	activit*.mp. [mp=abstract, title, original title, broad terms, heading words, identifiers, cabicodes]	1410700
39	exp space requirements/ or animal welfare/ or cage density/ or cage size/ or spacing/	62624
40	space*.mp. [mp=abstract, title, original title, broad terms, heading words, identifiers, cabicodes]	157273
41	human animal relationship*.mp. [mp=abstract, title, original title, broad terms, heading words, identifiers, cabicodes]	1273

42	play*.mp. [mp=abstract, title, original title, broad terms, heading words, identifiers, cabicodes]	378371
43	stimulat*.mp. [mp=abstract, title, original title, broad terms, heading words, identifiers, cabicodes]	248202
44	posture.mp. [mp=abstract, title, original title, broad terms, heading words, identifiers, cabicodes]	4223
45	(wellbeing or well-being).mp. [mp=abstract, title, original title, broad terms, heading words, identifiers, cabicodes]	29025
46	group size.mp. [mp=abstract, title, original title, broad terms, heading words, identifiers, cabicodes]	2527
47	human interaction*.mp. [mp=abstract, title, original title, broad terms, heading words, identifiers, cabicodes]	916
48	11 or 12 or 13 or 14 or 15 or 16 or 17 or 18 or 19 or 20 or 21 or 22 or 23 or 24 or 25 or 26 or 27 or 28 or 29 or 30 or 31 or 32 or 33 or 34 or 35 or 36 or 37 or 38 or 39 or 40 or 41 or 42 or 43 or 44 or 45 or 46 or 47	3653345
49	10 and 48	408
50	limit 49 to journal article	363
51	limit 50 to english language	340

1.3. Converted Search Strategy Syntax for each Database Searched

1. MEDLINE (OVID)   NEED TO UPDATE NUMBERS

Date of Final Search: 14/05/2022

Number of results: 642

Name: FULL SEARCH MEDLINE FINAL V5_CAPTIVE_CHIMP

#	Search string	# of results
1	Animals, Zoo/	5780
2	captiv*.mp. [mp=title, abstract, original title, name of substance word, subject heading word, floating sub-heading word, keyword heading word, organism supplementary concept word, protocol supplementary concept word, rare disease supplementary concept word, unique identifier, synonyms]	16071
3	zoo*.mp. [mp=title, abstract, original title, name of substance word, subject heading word, floating sub-heading word, keyword heading word, organism supplementary concept word, protocol supplementary concept word, rare disease supplementary concept word, unique identifier, synonyms]	62829
4	sanctuar*.mp. [mp=title, abstract, original title, name of substance word, subject heading word, floating sub-heading word, keyword heading word, organism supplementary	1695

	concept word, protocol supplementary concept word, rare disease supplementary concept word, unique identifier, synonyms]	
5	Pan troglodytes/	9889
6	chimpanzee*.mp. [mp=title, abstract, original title, name of substance word, subject heading word, floating sub-heading word, keyword heading word, organism supplementary concept word, protocol supplementary concept word, rare disease supplementary concept word, unique identifier, synonyms]	10631
7	pan troglodytes.mp. [mp=title, abstract, original title, name of substance word, subject heading word, floating sub-heading word, keyword heading word, organism supplementary concept word, protocol supplementary concept word, rare disease supplementary concept word, unique identifier, synonyms]	10205
8	1 or 2 or 3 or 4	76884
lih	5 or 6 or 7	13512
10	8 and 9	1109
11	Animal Welfare/	13653
12	welfare.mp. [mp=title, abstract, original title, name of substance word, subject heading word, floating sub-heading word, keyword heading word, organism supplementary concept word, protocol supplementary concept word, rare disease supplementary concept word, unique identifier, synonyms]	76876
13	stress*.mp. [mp=title, abstract, original title, name of substance word, subject heading word, floating sub-heading word, keyword heading word, organism supplementary concept word, protocol supplementary concept word, rare disease supplementary concept word, unique identifier, synonyms]	983052
14	Stress, Physiological/ or Stress, Psychological/	207425
15	health*.mp. [mp=title, abstract, original title, name of substance word, subject heading word, floating sub-heading word, keyword heading word, organism supplementary concept word, protocol supplementary concept word, rare disease supplementary concept word, unique identifier, synonyms]	3758548
16	"Quality of Life"/	232729
17	("quality of life" or quality-of-life).mp. [mp=title, abstract, original title, name of substance word, subject heading word, floating sub-heading word, keyword heading word, organism supplementary concept word, protocol supplementary concept word, rare disease supplementary concept word, unique identifier, synonyms]	346674
18	enrichment.mp. [mp=title, abstract, original title, name of substance word, subject heading word, floating sub-heading word, keyword heading word, organism supplementary concept word, protocol supplementary concept word, rare disease supplementary concept word, unique identifier, synonyms]	88580
19	Animal Husbandry/	22180
20	husbandry.mp. [mp=title, abstract, original title, name of substance word, subject heading word, floating sub-heading word, keyword heading word, organism supplementary concept word, protocol supplementary concept word, rare disease supplementary concept word, unique identifier, synonyms]	25580

21	exp Behavior, Animal/	297302
22	behavio*.mp. [mp=title, abstract, original title, name of substance word, subject heading word, floating sub-heading word, keyword heading word, organism supplementary concept word, protocol supplementary concept word, rare disease supplementary concept word, unique identifier, synonyms]	1575141
23	exp Aggression/	41682
24	aggress*.mp. [mp=title, abstract, original title, name of substance word, subject heading word, floating sub-heading word, keyword heading word, organism supplementary concept word, protocol supplementary concept word, rare disease supplementary concept word, unique identifier, synonyms]	211531
25	injur*.mp. [mp=title, abstract, original title, name of substance word, subject heading word, floating sub-heading word, keyword heading word, organism supplementary concept word, protocol supplementary concept word, rare disease supplementary concept word, unique identifier, synonyms]	1194864
26	wound*.mp. [mp=title, abstract, original title, name of substance word, subject heading word, floating sub-heading word, keyword heading word, organism supplementary concept word, protocol supplementary concept word, rare disease supplementary concept word, unique identifier, synonyms]	389416
meth27	(f?ecal adj3 glucocorticoid adj3 metabolite*).mp. [mp=title, abstract, original title, name of substance word, subject heading word, floating sub-heading word, keyword heading word, organism supplementary concept word, protocol supplementary concept word, rare disease supplementary concept word, unique identifier, synonyms]	219
28	Grooming/	3310
29	groom*.mp. [mp=title, abstract, original title, name of substance word, subject heading word, floating sub-heading word, keyword heading word, organism supplementary concept word, protocol supplementary concept word, rare disease supplementary concept word, unique identifier, synonyms]	7279
30	stereotyp*.mp. [mp=title, abstract, original title, name of substance word, subject heading word, floating sub-heading word, keyword heading word, organism supplementary concept word, protocol supplementary concept word, rare disease supplementary concept word, unique identifier, synonyms]	37093
31	mutilation.mp. [mp=title, abstract, original title, name of substance word, subject heading word, floating sub-heading word, keyword heading word, organism supplementary concept word, protocol supplementary concept word, rare disease supplementary concept word, unique identifier, synonyms]	6011
32	complexity.mp. [mp=title, abstract, original title, name of substance word, subject heading word, floating sub-heading word, keyword heading word, organism supplementary concept word, protocol supplementary concept word, rare disease supplementary concept word, unique identifier, synonyms]	135650
33	enclosure.mp. [mp=title, abstract, original title, name of substance word, subject heading word, floating sub-heading word, keyword heading word, organism supplementary concept word, protocol supplementary concept word, rare disease supplementary concept word, unique identifier, synonyms]	2461
34	relocat*.mp. [mp=title, abstract, original title, name of substance word, subject heading word, floating sub-heading word, keyword heading word, organism supplementary concept word, protocol supplementary concept word, rare disease supplementary concept word, unique identifier, synonyms]	10047

35	affiliative.mp. [mp=title, abstract, original title, name of substance word, subject heading word, floating sub-heading word, keyword heading word, organism supplementary concept word, protocol supplementary concept word, rare disease supplementary concept word, unique identifier, synonyms]	1489
36	activit*.mp. [mp=title, abstract, original title, name of substance word, subject heading word, floating sub-heading word, keyword heading word, organism supplementary concept word, protocol supplementary concept word, rare disease supplementary concept word, unique identifier, synonyms]	3179312
37	space*.mp. [mp=title, abstract, original title, name of substance word, subject heading word, floating sub-heading word, keyword heading word, organism supplementary concept word, protocol supplementary concept word, rare disease supplementary concept word, unique identifier, synonyms]	397657
38	human animal relationship*.mp. [mp=title, abstract, original title, name of substance word, subject heading word, floating sub-heading word, keyword heading word, organism supplementary concept word, protocol supplementary concept word, rare disease supplementary concept word, unique identifier, synonyms]	131
39	play*.mp. [mp=title, abstract, original title, name of substance word, subject heading word, floating sub-heading word, keyword heading word, organism supplementary concept word, protocol supplementary concept word, rare disease supplementary concept word, unique identifier, synonyms]	1154773
40	stimulat*.mp. [mp=title, abstract, original title, name of substance word, subject heading word, floating sub-heading word, keyword heading word, organism supplementary concept word, protocol supplementary concept word, rare disease supplementary concept word, unique identifier, synonyms]	1326282
41	posture.mp. [mp=title, abstract, original title, name of substance word, subject heading word, floating sub-heading word, keyword heading word, organism supplementary concept word, protocol supplementary concept word, rare disease supplementary concept word, unique identifier, synonyms]	79913
42	(wellbeing or well-being).mp. [mp=title, abstract, original title, name of substance word, subject heading word, floating sub-heading word, keyword heading word, organism supplementary concept word, protocol supplementary concept word, rare disease supplementary concept word, unique identifier, synonyms]	93545
43	group size.mp. [mp=title, abstract, original title, name of substance word, subject heading word, floating sub-heading word, keyword heading word, organism supplementary concept word, protocol supplementary concept word, rare disease supplementary concept word, unique identifier, synonyms]	2324
44	human interaction*.mp. [mp=title, abstract, original title, name of substance word, subject heading word, floating sub-heading word, keyword heading word, organism supplementary concept word, protocol supplementary concept word, rare disease supplementary concept word, unique identifier, synonyms]	1502
45	11 or 12 or 13 or 14 or 15 or 16 or 17 or 18 or 19 or 20 or 21 or 22 or 23 or 24 or 25 or 26 or 27 or 28 or 29 or 30 or 31 or 32 or 33 or 34 or 35 or 36 or 37 or 38 or 39 or 40 or 41 or 42 or 43 or 44	11107565
46	10 and 45	667

3. WEB OF SCIENCE CORE COLLECTION (WOS)

Date of Final Search: 15/04/2022

Number of results: 1454

#	Search string	# of results
1	((TS=(captiv*)) OR TS=(zoo*)) OR TS=(sanctuar*)	187532
2	(TS=(chimpanzee*)) OR TS=(pan troglodytes)	22723
3	((((((((((((((((((((((((((TS=(welfare)) OR TS=(stress*)) OR TS=(health*)) OR TS=("quality of life" OR quality-of-life)) OR TS=(enrichment)) OR TS=(husbandry)) OR TS=(behavio*)) OR TS=(aggress*)) OR TS=(injur*)) OR TS=(wound*)) OR TS=(f$ecal near/3 glucocorticoid near/3 metabolite*)) OR TS=(groom*)) OR TS=(stereotyp*)) OR TS=(mutilation)) OR TS=(complexity)) OR TS=(enclosure)) OR TS=(relocat*)) OR TS=(affiliative)) OR TS=(activit*)) OR TS=(space*)) OR TS=("human animal relationship*")) OR TS=(play*)) OR TS=(stimulat*)) OR TS=(posture)) OR TS=(wellbeing OR well-being)) OR TS=("group size")) OR TS=("human interaction*")	19438878
4	#1 AND #2 AND #3	1730
5	limited 4 to articles	1465
6	Limited 5 to English language	1454

4. BIOSIS CITATION INDEX (WOS)

Date of Final Search: 13/02/2022

Number of results: 1269

#	Search string	# of results
1	(((MC=(wildlife management OR Wildlife Management)) OR TS=(captiv*)) OR TS=(zoo*)) OR TS=(sanctuar*)	534928
2	(TS=(chimpanzee*)) OR TS=(pan troglodytes)	23098
3	((((((((((((((((((((((((((((((((TS=(welfare)) OR CC=(12008)) OR TS=(stress*)) OR MC=(veterinary medicine OR Veterinary Medicine)) OR TS=(health*)) OR TS=("quality of life" or quality-of-life)) OR TS=(enrichment)) OR MC=(Animal Care OR Animal Husbandry)) OR TS=(husbandry)) OR MC=(Behavior)) OR CC=(07003)) OR TS=(behavio*)) OR TS=(aggress*))) OR TS=(injur*)) OR TS=(wound*)) OR TS=(f$ecal near/3 glucocorticoid near/3 metabolite*)) OR TS=(groom*)) OR TS=(stereotyp*)) OR TS=(mutilation)) OR TS=(complexity)) OR TS=(enclosure)) OR TS=(relocat*)) OR TS=(affiliative)) OR TS=(activit*)) OR TS=(space*)) OR TS=("human animal relationship*")) OR TS=(play*)) OR TS=(stimulat*)) OR TS=(posture)) OR TS=(wellbeing OR well-being)) OR TS=("group size")) OR TS=("human interaction*")	13082020
4	#1 AND #2 AND #3	1860
5	limited 4 to articles	1296
6	limited 5 to English language	1269

5. ZOOLOGICAL RECORD (WOS)

Date of Final Search: 15/04/2022

Number of results: 1110

#	Search string	# of results
1	(((SD=(zoo OR Zoological gardens OR Zoos "AND" wildlife parks)) OR TS=(captiv*)) OR TS=(zoo*)) OR TS=(sanctuar*)	1485952
2	(TS=(chimpanzee*)) OR TS=(pan troglodytes)	8161
3	(((((((((((((((((((((((((((((((((TS=(welfare)) OR SD=(Physiological condition)) OR TS=(stress*)) OR SD=(Diseases "AND" disorders)) OR TS=(health*)) OR TS=("quality of life" or quality-of-life)) OR TS=(enrichment)) OR SD=(Care in captivity OR Diet in captivity OR Housing techniques)) OR TS=(husbandry)) OR SD=(Behaviour OR Abnormal behaviour)) OR TS=(behavio*)) OR SD=(Aggressive behaviour OR Agonistic behaviour OR Fighting)) OR TS=(aggress*)) OR TS=(injur*)) OR TS=(wound*)) OR SD=(Hormones OR Neuroendocrine system OR Endocrinology)) OR TS=(f?ecal near/3 glucocorticoid near/3 metabolite*)) OR TS=(groom*)) OR TS=(stereotyp*)) OR TS=(mutilation)) OR TS=(complexity)) OR TS=(enclosure)) OR SD=(Release "AND" relocation programmes OR Conservation measures)) OR TS=(relocat*)) OR TS=(affiliative)) OR TS=(activit*)) OR TS=(space*)) OR TS=("human animal relationship*")) OR TS=(play*)) OR TS=(stimulat*)) OR TS=(posture)) OR TS=("wellbeing" or well-being)) OR TS=("group size")) OR TS=("human interaction*")	1495544
4	#1 AND #2 AND #3	1312
5	limited 4 to articles	1174
6	limited 5 to English language	1110

6. SCOPUS

Date of Final Search: 15/04/2022

Number of results: 403

#	Search string	# of results
1	TITLE-ABS-KEY (captiv* OR zoo* OR sanctuar*)	254479

2	TITLE-ABS-KEY (chimpanzee* OR "pan troglodytes")	19227
3	(TITLE-ABS-KEY (welfare OR stress* OR health* OR "quality of life" OR enrichment OR husbandry OR behavio* OR aggress* OR injur* OR wound* OR f#ecal W/3 glucocorticoid W/3 metabolite* OR groom* OR stereotyp* OR mutilation OR complexity OR enclosure) OR TITLE-ABS-KEY (relocat* OR affiliative OR activit* OR space* OR "human animal relationship*" OR play* OR stimulat* OR posture OR (wellbeing OR (Quadros et al.)) OR "group size" OR "human interaction*"))	13,554,320
4	#1 AND #2 AND #3	462
5	limited 4 to articles	407
6	limited 5 to English language	403
